# Clinical outcomes of closed reduction vs. small-incision-assisted open reduction with intramedullary nailing in complex comminuted femoral shaft fractures (AO/OTA 32-C): a retrospective cohort study

**DOI:** 10.3389/fsurg.2025.1550063

**Published:** 2025-05-27

**Authors:** Qingwei Li, Jianqiang Wang, Chunyan Sun, Lintao Lu, Zongyou Mu, Xubin Zhang

**Affiliations:** ^1^ School of Clinical Medicine, Shandong Second Medical University, Weifang, China; ^2^ Department of Orthopedic, Qilu Hospital Dezhou Hospital of Shandong University Dezhou Hospital, Dezhou, Shandong, China

**Keywords:** femoral shaft fracture, intramedullary nailing, closed reduction, small-incision-assisted open reduction, AO/OTA 32-C, fracture healing, surgical outcomes

## Abstract

**Background:**

Intramedullary nailing (IMN) is the preferred treatment owing to its minimally invasive nature, high healing rates, and reduced stress shielding. However, the optimal reduction method for complex comminuted fractures (AO/OTA 32-C) has been controversial. Closed reduction preserves blood supply but requires extensive fluoroscopy and technical expertise. Small-incision-assisted open reduction enhances visualisation and facilitates reduction but entails slightly increased soft tissue exposure.

**Methods:**

This retrospective cohort study analysed 70 patients with AO/OTA 32-C femoral shaft fractures treated with intramedullary nailing. Patients were categorised into a Closed reduction group (*n* = 35) and Small-incision-assisted open reduction group (*n* = 35). Outcomes assessed included operative time, fluoroscopy usage, blood loss, infection rates, hospital stay duration, and functional outcomes at 3, 6, and 12 months postoperatively.

**Results:**

The Small-incision-assisted open reduction group had shorter operative times (45.09 ± 5.67 vs. 78.34 ± 5.71 min, *P* < 0.05) and lower fluoroscopy usage (6.03 ± 1.51 vs. 22.33 ± 5.99, *P* < 0.05). While blood loss and incision length were higher, infection rates and hospital stays were comparable between the groups. Functional outcomes at 3 and 6 months were significantly better in the Small-incision-assisted open reduction group, with no differences at 12 months. The Small-incision-assisted open reduction group also had a higher excellent-to-good fracture healing rate (88.6% vs. 60.0%, *P* < 0.05).

**Conclusions:**

Small-incision-assisted open reduction reduces operative time, fluoroscopy usage, and improves early functional outcomes. It is a safe and efficient alternative to closed reduction, but larger multi-centre studies are needed for broader validation.

## 1 Introduction

Femoral shaft fractures are a prevalent type of fractures, accounting for approximately 3.5% of all fractures (1, 2). These injuries are typically caused by high-energy trauma, such as direct impact, compression, or falls, and often result in wedge or comminuted fracture patterns. The nature of these fractures frequently involves significant soft tissue and vascular damage, making their reduction and treatment particularly challenging. Without appropriate treatment, complications such as limb shortening, delayed union, non-union, or permanent disability may arise, severely impacting patients' quality of life (3–5). Intramedullary nailing (IMN) has become the gold standard for treating femoral shaft fractures owing to its numerous advantages, including minimal invasiveness, reduced stress shielding, and high fracture-healing rates (6, 7). Despite these benefits, the optimal reduction method for managing complex comminuted femoral shaft fractures (AO/OTA 32-C) during IMN fixation remains a contentious issue.

Some researchers advocate for the use of closed reduction during IMN, emphasizing its potential to preserve the blood supply to already compromised tissues, minimise additional trauma, and reduce the risk of complications, thereby facilitating faster postoperative recovery (8). Others call for the adoption of Small-incision-assisted open reduction, highlighting its advantages in providing better surgical visualization, improved fracture-end reduction, shorter operative time, and more accurate alignment, despite the slightly increased soft tissue exposure and associated risks (9–11). Based on this background, this study retrospectively evaluates and compares the clinical outcomes of closed reduction and Small-incision-assisted open reduction during IMN fixation for complex comminuted femoral shaft fractures. This study aimed to compare the clinical outcomes, functional recovery, and complication between closed reduction and Small-incision-assisted open reduction in the treatment of complex femoral shaft fractures (AO/OTA 32-C) using intramedullary nailing.

## 2 Methods

### 2.1 Inclusion and exclusion criteria

#### 2.1.1 Inclusion criteria

Patients with radiographically confirmed comminuted femoral shaft fractures (AO/OTA 32-C); those undergoing antegrade IMN fixation; those without associated injuries or chronic diseases; and those with complete follow-up data for a minimum of 12 months were included.

#### 2.1.2 Exclusion criteria

Patients with severe comorbidities or pathological fractures deemed unsuitable for IMN fixation; those treated with plate fixation or external fixation; those with pre-existing limb dysfunction; those with coagulation disorders or significant psychiatric conditions; those with incomplete follow-up data.

### 2.2 Collection of patient data

This retrospective study was conducted based on the review of medical records from the hospital information system (HIS) and imaging archives of Qilu Hospital Dezhou Hospital of Shandong University. The data collection process followed standardised procedures to ensure consistency and accuracy.

The following data were extracted:

#### 2.2.1 Demographic and baseline information

Age, sex, time from injury (days), injury mechanism (e.g., traffic accident, fall, crush injury), and AO/OTA fracture classification.

#### 2.2.2 Perioperative clinical data

Surgical method (closed reduction or Small-incision-assisted open reduction), operation time (minutes), intraoperative blood loss (mL), number of intraoperative fluoroscopy images, incision length (cm), type of intramedullary nail used, length of hospital stay (days), and occurrence of intra- or postoperative complications (e.g., infection, nonunion, hardware failure).

#### 2.2.3 Postoperative follow-up data

Time to partial and full weight-bearing (days/weeks), functional outcomes assessed by Harris Hip Score (HHS) and HSS Knee Score at 3, 6, and 12 months, radiographic evidence of fracture healing (evaluated according to the Johner-Wruhs criteria), and any reoperation or revision procedures. All data were independently collected and cross-verified by two orthopaedic researchers to ensure accuracy and consistency. Missing or ambiguous information was clarified by reviewing imaging data and operative notes. Patients with incomplete records were excluded from the final analysis.

### 2.3 General clinical data

This retrospective cohort study included 70 patients with AO/OTA 32-C2/C3 femoral shaft fractures who underwent IMN fixation at our institution between January 2020 and December 2021, selected based on the inclusion and exclusion criteria. Patient data were obtained from medical records and surgical notes. All procedures were performed by a single senior orthopaedic surgeon with extensive experience in IMN fixation, ensuring consistency in surgical technique and minimizing variability. Patients were retrospectively classified into the closed reduction or Small-incision-assisted open reduction groups based on the surgical approach documented in operative records: Small-incision-assisted open reduction group (*n* = 35) and Closed reduction group (*n* = 35), the final distribution of 35 cases per group resulted naturally from retrospective case selection, without pre-determined numerical balancing or case matching. No selective inclusion or exclusion was performed to equalise group sizes. This study was conducted in accordance with the ethical standards of the Declaration of Helsinki and was approved by the Institutional Medical Ethics Committee. Informed consent for the use of anonymised clinical data was obtained from all patients and/or their legal guardians prior to inclusion in this study.

### 2.4 Surgical methods

#### 2.4.1 Closed reduction group

Patients in the Closed reduction group underwent closed reduction followed by antegrade IMN fixation. After combined spinal-epidural anaesthesia, patients were positioned supine on a traction table, with the unaffected limb abducted and the affected limb adducted for traction and reduction. Displacement and overlapping fracture fragments were corrected, and reduction quality was verified using C-arm fluoroscopy. A straight 4–5 cm incision was made along the lateral thigh, perpendicular to a line extending from the anterior superior iliac spine. The gluteus medius was bluntly dissected to expose the entry point at the apex of the greater trochanter. The surgeon manually compressed the proximal and distal fracture fragments or used instruments such as a “golden finger” to reach the distal fragment. After satisfactory reaming of the medullary cavity, the main nail was inserted, followed by fixation with interlocking screws. Reduction quality was reassessed under fluoroscopy before wound irrigation, closure, and the application of a sterile dressing.

#### 2.4.2 Small-incision reduction group

Patients in the Small-incision group underwent small-incision-assisted open reduction combined with interlocking IMN fixation. The proximal entry point and incision were identical to those used in the Closed reduction group. Following insertion of the guidewire into the medullary cavity, a 3 cm incision was made at the fracture site. Blunt dissection of the iliotibial band and vastus lateralis muscle allowed palpation of the fracture ends. Under manual traction provided by an assistant, the surgeon guided the wire into the distal fragment, ensuring proper reduction and alignment. Rotational alignment was verified using the femoral cortical line as a reference. Once provisional reduction was achieved, reaming and nail insertion were performed. Reduction quality was confirmed under fluoroscopy, and interlocking screws were inserted. The wound was then irrigated, closed, and dressed with sterile bandages.

### 2.5 Postoperative management

Both groups received standardised postoperative care, including infection prevention measures and venous thromboembolism prophylaxis. Regular follow-up x-rays were conducted to assess fracture healing progress. On the first postoperative day, patients were encouraged to perform ankle pump exercises and straight-leg raises to prevent thrombosis and promote recovery. Gradual weight-bearing was initiated depending on the stability and healing status of the fracture. Full weight-bearing was allowed only after radiographic evidence of callus formation. All patients were followed up at 1, 3, 6, and 12 months postoperatively for clinical and radiographic evaluation. All patients received the same type of antegrade interlocking femoral intramedullary nail (WEGO®, Shandong Weigao Orthopaedic Device Co., Ltd., China) and were initially treated with static locking to ensure consistency across both groups. Dynamic fixation (removal of distal locking screws) was considered only in cases of delayed union or insufficient callus formation during follow-up.

### 2.6 Observational indicators

Operative Time: Defined as the duration from skin incision to wound closure; Fluoroscopy Instances: The number of C-arm x-ray fluoroscopies performed intraoperatively; Intraoperative Blood Loss: Calculated as the blood absorbed by gauze combined with the volume collected by suction devices; Total Incision Length: Measured as the combined length of all surgical incisions, including the primary incision for intramedullary nail insertion, additional small incisions in the Small-incision group, and the small incisions made for interlocking screw placement; Length of Hospital Stay: Total days of hospitalization post-surgery; Infection Rates: The incidence of postoperative infections; Functional Outcomes: Evaluated using the Harris Hip Score (HHS) and the HSS Knee Score at designated postoperative time points; Fracture Healing Quality: Assessed using the Johner-Wruhs criteria (12): Excellent: Normal joint motion, normal gait, no pain, no angulation, shortening <5 mm, rotation <5°, no infection, or neurovascular complications; Good: Joint motion >75% of normal, slight strength limitation, occasional pain, angulation <5°, shortening 5–10 mm, rotation 5–10°, mild complications; Fair: Joint motion >50% of normal, significant weakness, moderate pain, angulation 10–20°, shortening 10–20 mm, rotation 10–20°, moderate complications; Poor: Delayed union or nonunion, joint motion <50%, inability to resist force, significant pain, angulation >20°, shortening >20 mm, rotation >20°, severe complications.

### 2.7 Statistical analysis

All data were analysed using SPSS version 25.0 statistical software. Continuous variables with a normal distribution are expressed as mean ± standard deviation (x¯ ± s), and comparisons between groups were performed using the independent sample t-test. For within-group comparisons at different postoperative time points, repeated measures analysis of variance (ANOVA, F-test) was utilised. Categorical data were expressed as counts and percentages, and intergroup comparisons were conducted using the chi-square test (*χ*^2^). Statistically significant *P*-values (*P* < 0.05) were highlighted in bold in the tables. A *P*-value <0.05 was considered statistically significant.

## 3 Results

### 3.1 Patient enrollment

**Figure 1:**
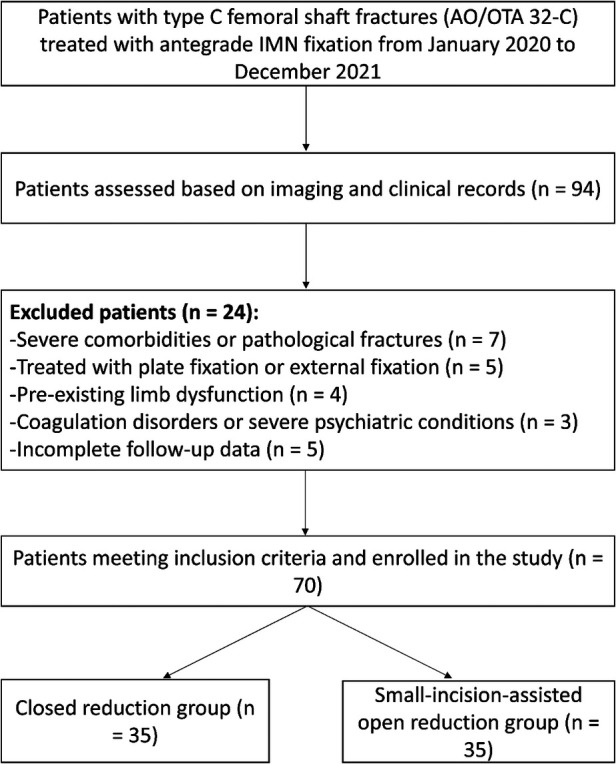
Case selection process of this retrospective study. Among 94 patients assessed between January and December 2021, 70 patients met the inclusion criteria divided into two.

All patients diagnosed with comminuted femoral shaft fractures (AO/OTA 32-C) between January 2020 and December 2021 were screened for eligibility. After applying the predefined inclusion and exclusion criteria, a total of 94 patients were identified. Of these, 24 were excluded owing to pathological fractures, severe comorbidities, use of alternative fixation methods, pre-existing limb dysfunction, coagulation disorders, or incomplete follow-up data. Ultimately, 70 patients met the inclusion criteria and were enrolled in the final analysis. The patient selection process is illustrated in Figure 1.

### 3.2 Baseline data

Baseline clinical characteristics, including gender, age, injury duration, mechanism of injury, and fracture classification, were compared between the two groups. No significant differences were observed between groups in these parameters, as shown in Table 1.

**Table 1:** Preoperative baseline characteristics of patients in the two groups.

Variable	Closed reduction group (*n* = 35)	Small-incision group (*n* = 35)	T/*χ*^2^* value	*P* value
Sex (male/female)	21/14	20/15	0.059*	0.810
Age (years)	54.11 ± 5.73	53.87 ± 5.69	0.864	0.185
Time from injury (days)	4.15 ± 0.93	4.12 ± 0.89	0.288	0.326
Fracture classification (C2/C3)	15/20	17/18	0.230*	0.632
Injury mechanism (Traffic/fall/crush/fall from height/other)	18/10/3/2/2	16/12/4/1/2	0.776*	0.941

* means χ² value.

### 3.3 Comparison of general surgical data between the two groups

A comparison of the general surgical data is presented in Table 2. Neither group experienced vascular or nerve injuries during the procedures. The Small-incision group had a significantly shorter operative time and fewer fluoroscopy instances than the Closed reduction group (*P* < 0.05). However, blood loss and total incision length were significantly greater in the Small-incision group (*P* < 0.05). No significant differences were observed between the two groups in infection rates or hospital stay duration (*P* > 0.05).

**Table 2:** Comparison of general surgical data between the two groups.

Variable	Closed reduction group (*n* = 35)	Small-incision group (*n* = 35)	T/χ^2^ *value	*P* value
Operative time (min)	78.3 ± 5.7	45.1 ± 5.7	5.039	**0**.**025**
Fluoroscopy times (*n*)	22.33 ± 5.99	6.03 ± 1.51	26.37	**0**.**013**
Blood loss (mL)	57.43 ± 13.61	88.57 ± 31.09	16.15	**0**.**039**
Total incision length (cm)	4.49 ± 0.43	7.37 ± 0.75	21.29	**0**.**013**
Length of hospital stay (days)	8.1 ± 2.3	8.6 ± 3.1	0.328	0.725
Infection (*n*, %)	1 (2.86%)	1 (2.86%)	0*	1.000

Bolded values mean statistically significant results.

* means χ² value.

### 3.4 Follow-up comparison between the two groups

Both groups were followed up for at least 12 months. The Small-incision group initiated weight-bearing and achieved full weight-bearing significantly earlier than the Closed reduction group (*P* < 0.05). Functional outcomes, as measured using Harris Hip Scores (HHS) and HSS Knee Scores, were improved significantly over time in both groups (*P* < 0.05). At 3 and 6 months postoperatively, the Small-incision group had significantly higher HHS and HSS scores than Closed reduction group (*P* < 0.05). By 12 months postoperatively, however, no significant differences were found between the two groups in either HHS or HSS scores. Detailed results are provided in Table 3.

**Table 3:** Follow-up outcomes between the two groups.

Indicator	Time point	Closed reduction group (*n* = 35)	Small-incision group (*n* = 35)	T value	*P* value
Start of weight-bearing (days)	–	48.73 ± 7.41	41.49 ± 5.68	10.485	**0.0024**
Full weight-bearing (weeks)	–	16.09 ± 4.63	14.85 ± 3.51	12.115	**0.020**
Harris hip score (HHS)	3 months	76.32 ± 6.11	80.21 ± 9.09	6.653	**0.041**
	6 months	80.50 ± 8.23	88.82 ± 9.32	10.208	**0.029**
	12 months	93.88 ± 2.52	95.95 ± 3.23	2.396	0.358
	*F* value	55.418	74.155	–	–
	*P* value	0.021	0.013	–	–
KSS knee score	3 months	65.41 ± 5.01	78.45 ± 6.18	7.454	**0.035**
	6 months	71.58 ± 5.30	80.25 ± 8.55	6.891	**0.023**
	12 months	87.18 ± 6.59	89.24 ± 8.54	2.997	0.238
	*F* value	53.226	46.413	–	–
	*P* value	0.027	0.037	–	–

Bolded values mean statistically significant results.

### 3.5 Comparison of complications

In the Closed reduction group, two patients experienced non-union, which was successfully addressed with secondary surgical intervention. Neither group reported cases of refracture, implant breakage, persistent non-union, or osteomyelitis. Fracture healing quality was assessed using the Johner-Wruhs criteria, a widely accepted classification system for long bone fracture outcomes. At the 12-month follow-up, 27 patients in the Small-incision group were classified as excellent, 4 as good, 3 as fair, and 1 as poor, resulting in an excellent-good rate of 88.6% (31/35). In the Closed reduction group, 15 patients were classified as excellent, 7 as good, 10 as fair, and 3 as poor, yielding an excellent-good rate of 60.0% (21/35). The Small-incision group exhibited a significantly lower complication rate and a higher excellent-good fracture healing rate than Closed reduction group did (*P* < 0.05). Detailed comparisons are shown in Table 4.

**Table 4:** Comparison of fracture healing quality between the two groups.

Group	Excellent	Good	Fair	Poor
Small-incision group	27	4	3	1
Closed reduction group	15	7	10	3
χ^2^ value	9.218	–	–	–
*P* value	**0.027**	–	–	–

Bolded values mean statistically significant results.

## 4 Discussion

The results of this study indicated that the minimally invasive assisted reduction group outperformed the Closed reduction group in several aspects: it had a shorter operative time, fewer intraoperative fluoroscopy exposures, earlier weight-bearing initiation, and better functional recovery at 3 and 6 months postoperatively. Although the functional outcomes between the two groups were no longer significantly different at 12 months after surgery, the fracture healing rate remained significantly higher in the minimally invasive group. These findings suggest that, in the treatment of complex comminuted femoral shaft fractures, minimally invasive assisted open reduction not only preserves the biomechanical advantages of IMN, but also improves surgical efficiency and clinical outcomes, making it a more effective and superior alternative.

Femoral shaft fractures, defined as those occurring between 5 cm below the lesser trochanter and 5 cm above the adductor tubercle, are common in clinical practice (13, 14). IMN is the standard treatment, offering stable fixation, stress distribution, and preservation of limb alignment (7, 9, 10, 15). Reamed IMN generates bone debris that promotes healing, and vascular disruption is typically repaired within six weeks (16). Open reduction in IMN procedures, though effective for anatomical alignment, often results in significant blood loss and a higher risk of infection. In contrast, closed reduction has become increasingly popular due to its ability to preserve blood supply, reduce soft tissue damage, and minimise intraoperative bleeding (17–19). Nonetheless, closed reduction demands higher technical expertise, often requires the use of a traction table, and relies on frequent intraoperative fluoroscopy to confirm proper fracture alignment. It places high demands on the surgeon's experience, leads to prolonged operative time, and increases radiation exposure. These factors pose challenges to its widespread adoption, especially in resource-limited clinical settings. The difficulty is further heightened in comminuted fractures, where rotational alignment is more complex, the procedure becomes more technically demanding, and the duration of surgery is extended (20, 21). In this study, the mean operative time for the Closed reduction group was 78.34 ± 5.71 min, which is consistent with previously reported durations for closed intramedullary nailing procedures in the literature (22, 23). This fully reflects the technical complexity of the procedure.

Against this background, minimally invasive assisted reduction has gradually attracted attention as an alternative approach that balances precise fracture reduction with minimised surgical trauma. In this study, a small incision of approximately 3 cm was made at the centre of the fracture site, and reduction was achieved through blunt dissection and tactile guidance. This technique preserves the periosteum and minimises soft tissue stripping while retaining the fracture hematoma and local biological environment, which are beneficial for bone healing. Additionally, bone debris generated during intramedullary reaming remains around the fracture site, serving a function similar to that of autologous bone grafting (24). Compared with closed reduction, which requires multiple fluoroscopic confirmations, the small-incision approach relies on direct tactile feedback, thereby improving reduction accuracy and significantly reducing the number of fluoroscopy exposures and intraoperative radiation—enhancing safety for both patients and surgeons. The results of this study also showed that the Small-incision group had a shorter operative time and required fewer fluoroscopy exposures. These findings are consistent with previous reports describing the difficulties of fracture reduction and frequent fluoroscopy in comminuted fractures treated with closed reduction, and they align with studies highlighting the time efficiency advantage of minimally invasive assisted reduction in such cases (20, 25–27).

Previous studies have reported no statistically significant differences in postoperative functional outcomes between minimally invasive assisted reduction and closed reduction, which contrasts with our findings (25). In our study, the minimally invasive assisted reduction group demonstrated significantly better Harris Hip Scores (HHS) and Hospital for Special Surgery (HSS) knee scores at 3 and 6 months postoperatively, suggesting a smoother rehabilitation process. This discrepancy may be attributed to differences in fracture severity: prior studies mainly included AO 32A1–B2 fractures, whereas our study focused on AO 32-C fractures, which are more complex and comminuted. These fractures place higher demands on the quality of reduction, and suboptimal reduction is more likely to impair postoperative functional recovery. Karaman et al. reported that, among patients treated with closed reduction and intramedullary nailing for femoral shaft fractures, computed tomography evaluation revealed rotational deformities greater than 10° in 41.7% of cases. These patients often presented with significant clinical symptoms and had markedly lower functional scores than those without deformities (28). In contrast, open reduction techniques enable more accurate anatomical alignment of the fracture ends, thereby reducing the risk of malalignment. Relevant meta-analyses have also shown that, compared with closed reduction, open reduction can reduce the risk of malunion to less than one-third (29). Although no significant difference was observed in functional scores between the two groups at 12 months postoperatively—indicating comparable long-term outcomes—the minimally invasive group exhibited better fracture healing quality and a significantly higher rate of excellent-to-good outcomes. This may be attributed to the improved accuracy of fracture reduction and the reduced disruption to surrounding soft tissues associated with the minimally invasive approach.

In terms of safety, the study found no significant differences between the minimally invasive assisted reduction group and the Closed reduction group in postoperative infection rates or incidences of neurovascular injury, indicating good safety profiles for both approaches. However, two cases of non-union occurred in the Closed reduction group, which may be attributed to the interposition of soft tissue within the fracture gap, hindering bone healing**.** In both non-union cases, dynamic fixation was implemented by removing the distal locking screws during follow-up to promote callus formation and enhance fracture healing. In contrast, the small-incision technique allows for direct visualisation and intraoperative removal of interposed tissue, thereby reducing the risk of such complications. Regarding postoperative infections, one case of wound infection occurred in each group, with an incidence rate of 2.9% (1/35), and both were well-controlled following antibiotic treatment. These infection rates fall within the range reported in the literature (1%–3.8%) (9, 25, 30), and no significant difference was observed between the two groups. This is consistent with previous studies and may be related to the minimally invasive nature of both procedures, the small incision size, limited intraoperative soft tissue disruption, and standardised postoperative infection management. Moreover, closed reduction carries risks such as perineal compression injuries, vascular or nerve damage, and fat embolism due to prolonged manipulation. In contrast, limited open reduction mitigates these risks by minimizing manipulation and reducing dependence on traction tables (31, 32).

This study has some limitations. First, its retrospective design may introduce inherent selection bias. Second, the sample size was relatively small, and all patients were treated at a single centre, which may limit the generalisability of the findings. Third, while surgical procedures were performed by experienced orthopaedic surgeons, variability in individual technique and intraoperative decision-making may have influenced outcomes. Last, the study did not include long-term follow-up beyond 12 months, which may be necessary to evaluate the durability of functional recovery and implant survival. Future multi-centre prospective studies with larger sample sizes and extended follow-up periods are warranted to validate and expand upon these results.

## 5 Conclusion

In conclusion, both closed reduction and small-incision-assisted open reduction combined with intramedullary nailing are effective treatment options for complex comminuted femoral shaft fractures (AO/OTA 32-C). However, the small-incision-assisted open reduction technique demonstrated distinct advantages in terms of shorter operative time, reduced fluoroscopy exposure, earlier initiation of weight-bearing, and superior functional recovery during the early postoperative period. These findings suggest that limited open reduction may be a more efficient and clinically beneficial approach for promoting timely fracture healing and early rehabilitation.

## Data Availability

The original contributions presented in the study are included in the article/Supplementary Material, further inquiries can be directed to the corresponding author.
